# Aspirin–Fisetin Combinatorial Treatment Exerts Cytotoxic and Anti-Migratory Activities in A375 Malignant Melanoma Cells

**DOI:** 10.3390/medicina60071125

**Published:** 2024-07-12

**Authors:** Claudia Iftode, Daliana Minda, George Draghici, Andreea Geamantan, Sorin Ursoniu, Ileana Enatescu

**Affiliations:** 1 Faculty of Medicine, “Victor Babeș” University of Medicine and Pharmacy from Timisoara, Eftimie Murgu Square No. 2, 300041 Timisoara, Romania; 2 Faculty of Pharmacy, “Victor Babes” University of Medicine and Pharmacy from Timisoara, Eftimie Murgu Square No. 2, 300041 Timisoara, Romania; 3 Research Center for Pharmaco-Toxicological Evaluations, Faculty of Pharmacy, “Victor Babes” University of Medicine and Pharmacy from Timisoara, Eftimie Murgu Square No. 2, 300041 Timisoara, Romania; 4 Center for Translational Research and Systems Medicine, “Victor Babes” University of Medicine and Pharmacy from Timisoara, Eftimie Murgu Square No. 2, 300041 Timisoara, Romania

**Keywords:** aspirin, fisetin, malignant melanoma, cytotoxicity, migration, irritant effect, angiogenesis

## Abstract

*Background and Objectives:* Malignant melanoma (MM) remains one of the most aggressive cancers worldwide, presenting a limited number of therapeutic options at present. Aspirin (ASA), a broadly used non-steroid anti-inflammatory medicine, has recently emerged as a candidate for repurposing in cancer management, due to its therapeutic potential in the treatment of several neoplasms which include MM. Fisetin (FIS) is a flavonoid phytoestrogen instilled with multispectral pharmacological activities, including a potent anti-melanoma property. The present study aimed to assess the potential improved anti-neoplastic effect resulting from the association of ASA and FIS for MM therapy. *Materials and Methods:* The study was conducted using the A375 cell line as an experimental model for MM. Cell viability was assessed via the MTT test. Cell morphology and confluence were evaluated using bright-field microscopy. The aspect of cell nuclei and tubulin fibers was observed through immunofluorescence staining. The irritant potential and the anti-angiogenic effect were determined on the chorioallantoic membrane of chicken fertilized eggs. *Results:* The main findings related herein demonstrated that the ASA 2.5 mM + FIS (5, 10, 15, and 20 µM) combination exerted a higher cytotoxicity in A375 MM cells compared to the individual compounds, which was outlined by the concentration-dependent and massive reduction in cell viability, loss of cell confluence, cell shrinkage and rounding, apoptotic-like nuclear features, constriction and disruption of tubulin filaments, increased apoptotic index, and suppressed migratory ability. ASA 2.5 mM + FIS 20 µM treatment lacked irritant potential on the chorioallantoic membrane and inhibited blood-vessel formation in ovo. *Conclusion:* These results stand as one of the first contributions presenting the anti-melanoma effect of the ASA + FIS combinatorial treatment.

## 1. Introduction

Cancers represent a complex and heterogeneous group of malignant tumors instilled with an uncontrollable spread and collectively contributing to a high rate of mortality in patients. Malignant melanoma (MM) stands as one of the most frequent cancer types diagnosed in adults, along with lung, prostate, and breast neoplasms, being an aggressive disease and a primary cause of premature cancer-related deaths [1,2,3]. According to a recent report, the incidence rate in Europe stands at 10–25 new MM cases per 100,000 inhabitants and is continuously rising [4]. MM is defined as a severe form of cancer that originates in the melanin-producing cells called melanocytes, as a consequence of various genetic or environmental risk factors (e.g., familial history of melanoma, pigmentary characteristics, sun exposure, etc.) [4], and is also deemed a malignancy with increased risk of systemic spread [5]. Currently, MM treatment implies surgical resection, photodynamic therapy, chemotherapy, immunotherapy, or targeted therapy, individually or in association, with the therapeutic strategy depending on the stage of the disease, the localization of the tumor, and the specific characteristics of the patient [6]. Nonetheless, many of the current established therapies against MM present vast limitations such as severe side effects, tumor resistance to the allocated treatment, or poor patient response rate [7] that diminish the life quality of the treated patients, lead to poorer survival outcomes [5], and raise the urgent need for innovative treatment modalities that will reduce these disadvantages, while also potentiating a higher success rate for oncological patients [8].

Over the last several decades, medicinal plants and their derived compounds have acquired outstanding interest due to their versatile properties. Natural products possess structural diversity and potential for multi-target effects, representing a substantial advancement for modern healthcare [9]. Due to the chemical diversity in nature, many botanical compounds are potential tools in cancer prevention or even cancer treatment, with various ongoing clinical trials testing their safety profile and efficacy in this regard [10]. Possessing a non-toxic profile and a pleiotropic mechanism of action, phytocompounds are considered promising candidates in combination with other agents such as chemotherapeutics as a strategy to increase effectiveness in the fight against tumors [11,12], also holding great potential for MM chemoprevention and treatment of melanoma [13]. One such natural product is fisetin (FIS), a flavonoid phytoestrogen with recognized anti-oxidant and anti-inflammatory effects that is widely distributed in vegetables and fruits [14]. A particular interest has been given recently to its anti-cancer properties, the mechanisms underlying the antitumor action of FIS being represented by its ability to target the cell cycle, invasion, and metastasis, as well as to promote apoptosis through elevation of pro-apoptotic Bax and caspase 3/8 [15]. In particular, by modulating various cell signaling pathways (e.g., apoptosis, inflammation, angiogenesis, etc.) and by enhancing the effectiveness of chemotherapy, FIS was described as a potential therapeutic candidate for the management of various cancers including MM [14,16,17]. FIS was previously found to inhibit the proliferation and invasion of MM cells by targeting the MAPK and NFkB pathways and promoting the mesenchymal-to-epithelial transition [17,18].

Drug repurposing, also known as drug repositioning, emerged as a novel low-cost drug development approach that aims at the discovery of new therapeutic applications for available, clinically approved, and marketed medicines, being extensively employed at present for the obtainment of new treatments for various cancers, including MM [18,19]. One of the earliest examples of drug repurposing is related to the indications of aspirin (ASA) [20], a non-steroid anti-inflammatory salicylate that yields a broad spectrum of pharmacological activities including antipyretic, analgesic, and antiplatelet properties [21]. Nowadays, numerous investigations on the anti-neoplastic activity of ASA have emerged [22], some studies in particular associating the chronic administration of ASA with the reduced risk of skin cancer development [23,24,25]. ASA was also found to exert anti-melanoma effects through different mechanisms of action, such as the suppression of colony formation and cell motility, the induction of mitochondrial toxicity, and the generation of reactive oxygen species (ROS) [25]. In addition to its intrinsic anti-cancer effects, ASA was also evaluated in association with a variety of drugs or natural compounds (e.g., exemestane, cisplatin, 5-fluorouracil, and genistein) as a potential improved therapeutic approach in cancer treatment, and the results were promising [11,26,27,28].

In light of these previously documented data, the present study proposed a preclinical assessment of the potential therapeutic efficacy of the ASA + FIS combinatorial treatment in MM management. Debuting with an in vitro evaluation of the cytotoxicity and anti-migratory effect of the ASA + FIS association in A375 MM cells, this research also provides an insight into its angio-suppressing property in ovo, contributing to further investigations in this regard.

## 2. Materials and Methods

### 2.1. Materials and Reagents

The evaluated substances (aspirin—ASA, and fisetin—FIS), Trypan blue 0.4%, DAPI, and MTT kit were obtained from Sigma Aldrich (St. Louis, MO, USA). All products used for cell culture (Dulbecco’s Modified Eagle Medium, trypsin-EDTA, dimethyl sulfoxide—DMSO, fetal bovine serum—FBS, and antibiotics) were delivered by ATCC (American Type Cell Collection, Lomianki, Poland). The reagents applied for immunofluorescence staining (Triton-X, alpha-tubulin monoclonal antibody (B-5-1-2), goat anti-mouse IgG (H + L) secondary antibody Alexa Fluor™ 488, paraformaldehyde 4%, and bovine serum albumin—BSA were obtained from Thermo Fisher Scientific Inc. (Waltham, MA, USA), Santa Cruz Biotechnology (Dallas, TX, USA), and Cell Signaling Technology (Danvers, MA, USA), respectively.

### 2.2. Instruments

The equipment used for the conducted experiments (the Cytation 5 plate reader, the Lionheart FX microscope, the Gen5™ Microplate Data Collection and Analysis Software 3.14, and the Autoscratch Wound Making Tool) were bought from BioTek Instruments Inc. (Winooski, VT, USA).

### 2.3. In Vitro Models and Cell Culture

A375 MM cells (CRL-1619 ™) and HaCaT keratinocytes (300493) were obtained from ATCC and CLS Cell Lines Service GmbH (Eppelheim, Germany) as frozen vials and cultured according to the manufacturer’s indications, presenting normal proliferation and shape during the experiments. The cells were grown in Dulbecco’s Modified Eagle Medium containing the required supplements (10% FBS, and 1% antibiotics) and in proper conditions (37 °C and 5% CO_2_).

### 2.4. Cytotoxicity Assessments

The effect of ASA, FIS, and ASA + FIS on the viability of A375 and HaCaT cells was investigated using the MTT kit, at 72 h following treatment. For this assay, the cells were plated in 96-well clear plates and stimulated with ASA, FIS, and ASA + FIS. Finally, MTT reagent was first added for 3 h at 37 °C and 5% CO_2_, followed by solubilization solution for 30 min at room temperature. The absorbance values were measured on Cytation 5 at 570 and 630 nm.

### 2.5. Bright-Field Analysis of Cell Shape and Confluence

The influence of ASA, FIS, and ASA + FIS on the shape and confluence of A375 cells after 72 h of treatment was determined by taking representative images of the treated cells in Bright-field, with the Lionheart FX microscope. All images were analyzed using the Gen5™ Microplate Data Collection and Analysis Software 3.14.

### 2.6. Immunofluorescence Visualization of Nuclei and Cytoskeletal Tubulin

The visualization of cellular components following ASA, FIS, and ASA + FIS treatments was performed via immunofluorescence staining after 72 h of exposure to the samples. Nuclei were counterstained with DAPI (1:500 dilution in 0.1% BSA) for five minutes at room temperature. Tubulin was observed after the cell’s treatment with alpha-tubulin monoclonal antibody (B-5-1-2) (1:500 dilution in 0.1% BSA) and goat anti-mouse IgG (H + L) secondary antibody (Alexa Fluor™ 488) (1:500 dilution in 0.1% BSA). Before these steps, the cells were cultured in 96-well black plates with clear bottoms, and treated with paraformaldehyde 4%, Triton X 0.1%, and BSA 1%, as detailed in a previous publication [29]. The images were obtained using the Lionheart FX microscope and the Gen5 ™ Microplate Data Collection and Analysis Software Version 3.14.

### 2.7. Cell Migration Assay

The influence of ASA, FIS, and ASA + FIS on the migration of A375 cells was assessed by performing the scratch assay. In brief, the cells were cultured in Corning Costar 24-well plates, an automatic scratch was made in each well using the AutoScratch™ Wound Making Tool, the cells were treated with ASA, FIS, and ASA + FIS for 24 h, and representative images (at magnification 4×) of the scratch area were taken at 0 and 24 h post-treatment on Cytation 1 (BioTek^®^ Instruments Inc., Winooski, VT, USA). The width of the performed scratches was measured in Gen5 ™ Microplate Data Collection and Analysis Software Version 3.14.

### 2.8. In Ovo Hen’s Egg Test–Chorioallantoic Membrane (HET–CAM) Test

The in ovo experiments were performed using embryonated chicken eggs prepared as described in a previous publication [30]. The irritation test for ASA + FIS was conducted on the tenth day of the eggs’ incubation, using a solution of 1% sodium dodecyl sulfate—SDS as a positive control, and H_2_O as a negative control. Simply, the samples were directly applied on the CAM and the appearance of hemorrhage (H), lysis (L), and coagulation (C) was followed for five minutes. Images were taken on the Discovery 8 SteREO microscope (Zeiss, Göttingen, Germany) at T0 and T5, time points indicating the beginning and the end of the experiment. Image processing was performed using the ZEN core version 3.8 software (Zeiss, Göttingen, Germany). Irritation score (IS) was calculated to assess the irritant effect of the tested compounds.

### 2.9. In Ovo Evaluation of Angiogenesis

The angiogenesis study was performed between the 8th and the 10th day of embryonic development, considering that at this point the CAM reaches the peak for neovascularization [31]. The protocol applied is similar to the one described by Parveen et al. [32]. In brief, the ASA 2.5 mM + FIS 20 µM solution (prepared in distilled water) was directly applied to cover the CAM. The eggs were next incubated for 24 h at 37 °C and 60% humidity. Finally, representative images were taken on the Discovery 8 SteREO microscope and processed using the ZEN core version 3.8 software. The IKOSA Prism Application CAM assay (v3.1.0) was employed to conduct a quantitative assessment of the vessels’ total area and number of vascular branching points.

### 2.10. Statistical Analysis

Statistical analysis of all data was performed on GraphPad Prism software version 10.2.3—GraphPad Software, San Diego, CA, USA, www.graphpad.com (accessed on 15 May 2024), and by employing two statistical methods: the one-way ANOVA analysis and Dunnett’s multiple-comparison test, respectively. All statistically significant results were marked using “*”, as follows: * *p* < 0.05; ** *p* < 0.01; *** *p* < 0.001; **** *p* < 0.0001.

## 3. Results

### 3.1. Cytotoxicity of ASA, FIS, and ASA + FIS

**Figure 1:**
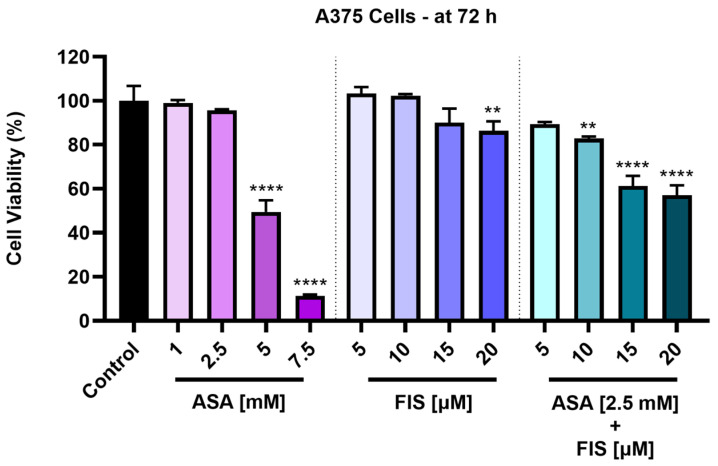
Viability of A375 cells treated for 72 h with aspirin (ASA), fisetin (FIS), and their association. The results were normalized to control (A375 cells without treatment) and represent means ± standard deviation of three experiments done as triplicates. One-way ANOVA analysis and Dunnett’s multiple-comparison post-test were performed to determine the statistically significant differences between control and treatment (** *p* < 0.01; **** *p* < 0.0001).

The cytotoxic effects of ASA, FIS, and their combinatorial treatment were first assessed in A375 MM cells after a 72 h treatment interval. As illustrated in Figure 1, all applied treatments exerted a concentration-dependent loss of viability in this cell line. ASA was not cytotoxic in A375 cells at the lowest concentrations tested of 1 mM and 2.5 mM, the viability percentages being 99.07% and 95.62%, respectively. However, a significant reduction in viability was caused by ASA 5 mM (to 49.47%) and 7.5 mM (to 11.35%). As regards the impact of FIS on A375 cells’ viability, the lowest concentrations (5 µM and 10 µM) showed a slight stimulatory effect, the viabilities increasing over 100%. The highest concentrations (15 µM and 20 µM) inhibited cell viability, which was lowered to 90.04% and 86.40%, respectively. Statistical significance was reached only for the highest concentration of FIS. ASA 2.5 mM enhanced the cytotoxicity of FIS (5, 10, 15, and 20 µM), the percentages of A375 cells being lower compared to the individual treatment with FIS or ASA 2.5 mM. Thus, the viabilities for the ASA 2.5 mM + FIS 5 µM, ASA 2.5 mM + FIS 10 µM, ASA 2.5 mM + FIS 15 µM, and ASA 2.5 mM + FIS 20 µM were 89.33%, 82.86%, 61.24%, and 57.09%, respectively.

**Figure 2:**
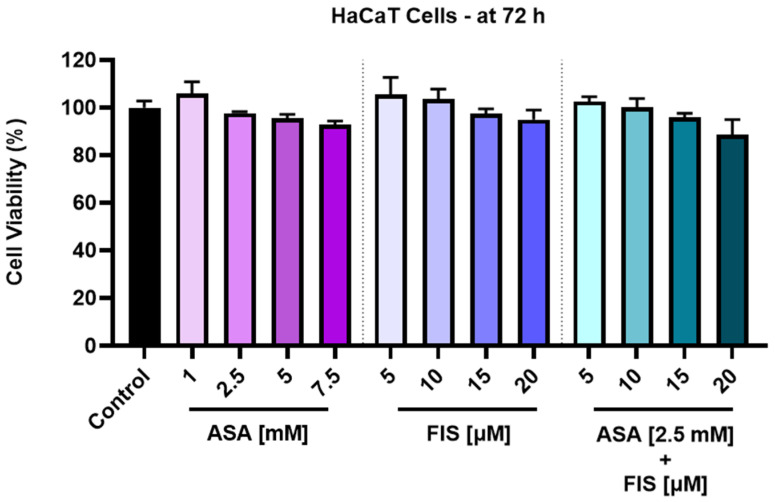
Viability of HaCaT keratinocytes treated for 72 h with aspirin (ASA), fisetin (FIS), and their association. The results were normalized to control (HaCaT keratinocytes without treatment) and represent means ± standard deviation of three experiments done as triplicates. One-way ANOVA analysis and Dunnett’s multiple-comparison post-test were performed to determine the statistically significant differences between control and treatment.

ASA, FIS, and ASA + FIS treatments showed selective cytotoxicity towards MM cells, exerting no effect on the viability of healthy skin-derived HaCaT keratinocytes, which was over 85% after the 72 h exposure (Figure 2).

### 3.2. Influence of ASA, FIS, and ASA + FIS on Cellular Morphology and Confluence

**Figure 3:**
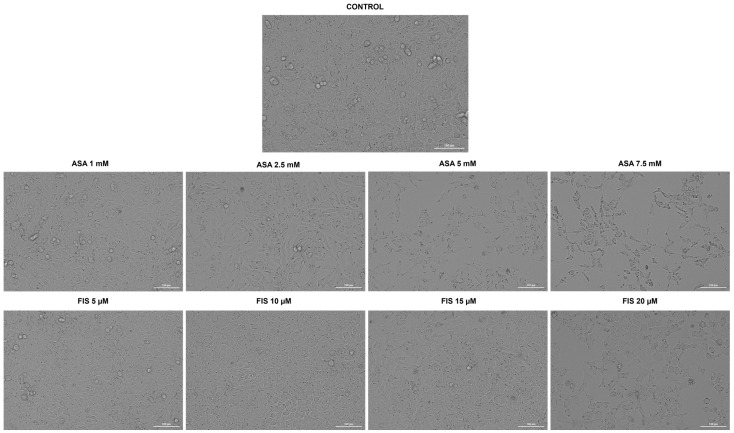
Morphology and confluence of A375 cells treated for 72 h with aspirin (ASA) and fisetin (FIS). Scale bars show 100 µm.

As part of their cytotoxic profile, the influence of ASA, FIS, and ASA + FIS on the shape and confluence of A375 cells was further verified. Figure 3 depicts the morphological changes that occurred in this cell line after a 72 h individual treatment with ASA (1, 2.5, 5, and 7.5 mM) and FIS (5, 10, 15, and 20 µM). At the lowest concentration of 1 mM, ASA had no impact of A375 cells’ morphology or confluence, while at 2.5 mM, it caused a slight reduction in confluence accompanied by cell elongation. At the highest concentrations of 5 mM and 7.5 mM, not only did the cells lose their confluence and adherence to neighboring cells, but they also became massively shrunken. As regards FIS, at 5 µM and 10 µM, A375 cells appeared more agglomerated compared to the control, while at 15 µM and 20 µM, a visible loss of confluence was detected. Shrinkage was also observed in A375 cells treated for 72 h with FIS 20 µM.

**Figure 4:**
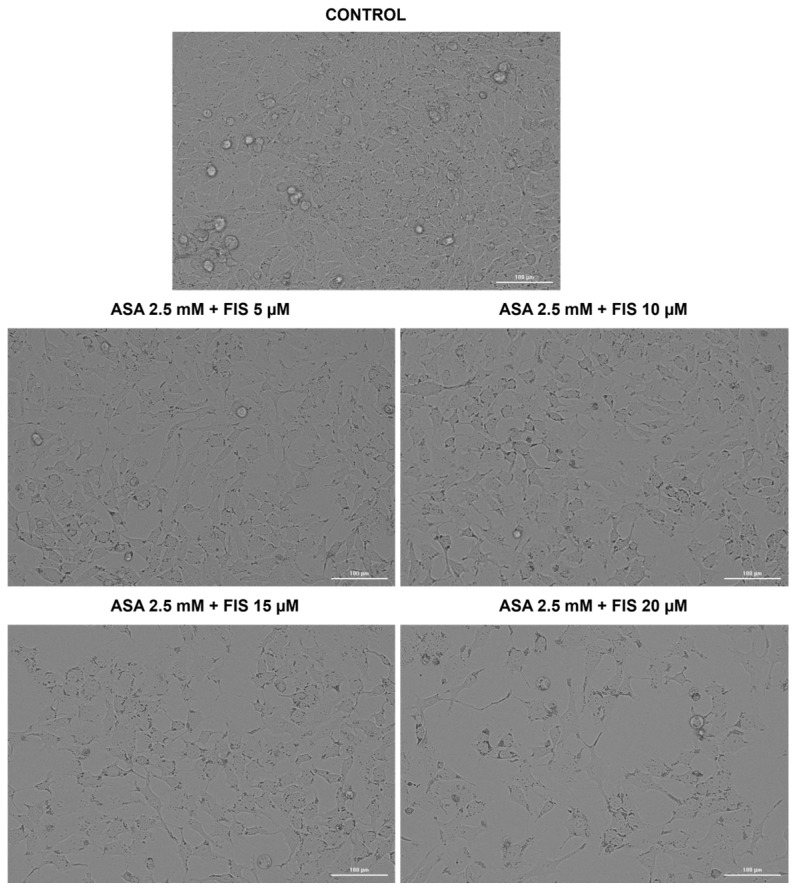
Morphology and confluence of A375 cells treated for 72 h with the combinatorial treatment between aspirin (ASA) and fisetin (FIS). Scale bars show 100 µm.

All combinatorial treatments comprising ASA 2.5 mM and FIS (5, 10, 15, and 20 µM) produced a reduction in A375 cells’ confluence; however, the most significant effect was obtained for ASA 2.5 mM + FIS 20 µM association, which additionally triggered changes in morphology such as shrinkage and rounding (Figure 4).

### 3.3. Influence of ASA, FIS, and ASA + FIS on Nuclear Shape and Tubulin Distribution

**Figure 5:**
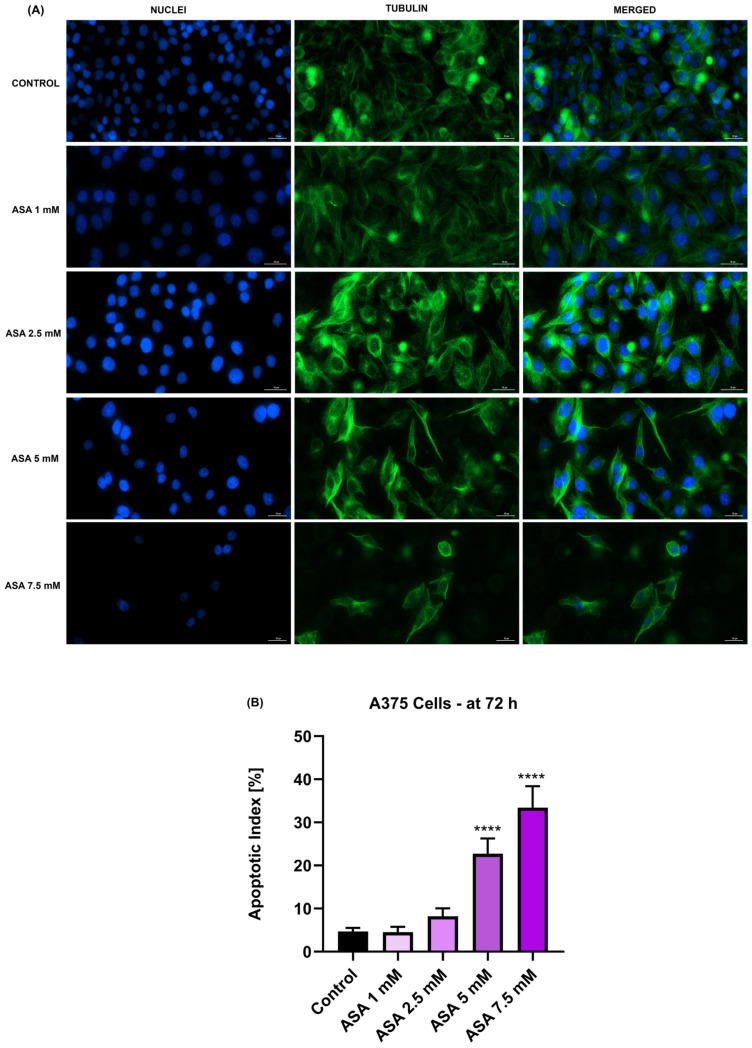
(**A**) Nuclei (blue) and tubulin filaments (green) of A375 cells treated for 72 h with aspirin (ASA) at four millimolar concentrations (1, 2.5, 5, and 7.5). Scale bars show 30 µm. (**B**) Apoptotic index calculation in A375 cells treated for 72 h with aspirin (ASA) at four milimolar concentrations (1, 2.5, 5, and 7.5 mM). The results represent means ± standard deviation of three experiments done as triplicates. One-way ANOVA analysis and Dunnett’s multiple-comparison post-test were performed to determine the statistically significant differences between control and treatment (**** *p* < 0.0001).

The aspect of cell nuclei and cytoskeletal tubulin filaments following the exposure of A375 cells for 72 h to ASA, FIS, and ASA + FIS was next investigated. As shown in Figure 5A, ASA 1 mM induced no alterations at the nuclear or cytoskeletal levels, the treated cells presenting similar features to those without treatment (control). Nonetheless, at a higher concentration (2.5 mM), visible constriction of nuclei and tubulin fibers was noticed. ASA 5 mM triggered chromatin condensation and nuclear dysmorphology, as well as tubulin constriction, alterations that were accompanied by a change in the shape of A375 cells, which became elongated as compared to control. At the highest concentration of 7.5 mM, apart from nuclear condensation and blebbing, ASA induced tubulin reorganization and cell rounding. A significant increase in the percentage of nuclei presenting apoptotic-like characteristics was obtained only for ASA 5 mM and 7.5 mM (Figure 5B).

**Figure 6:**
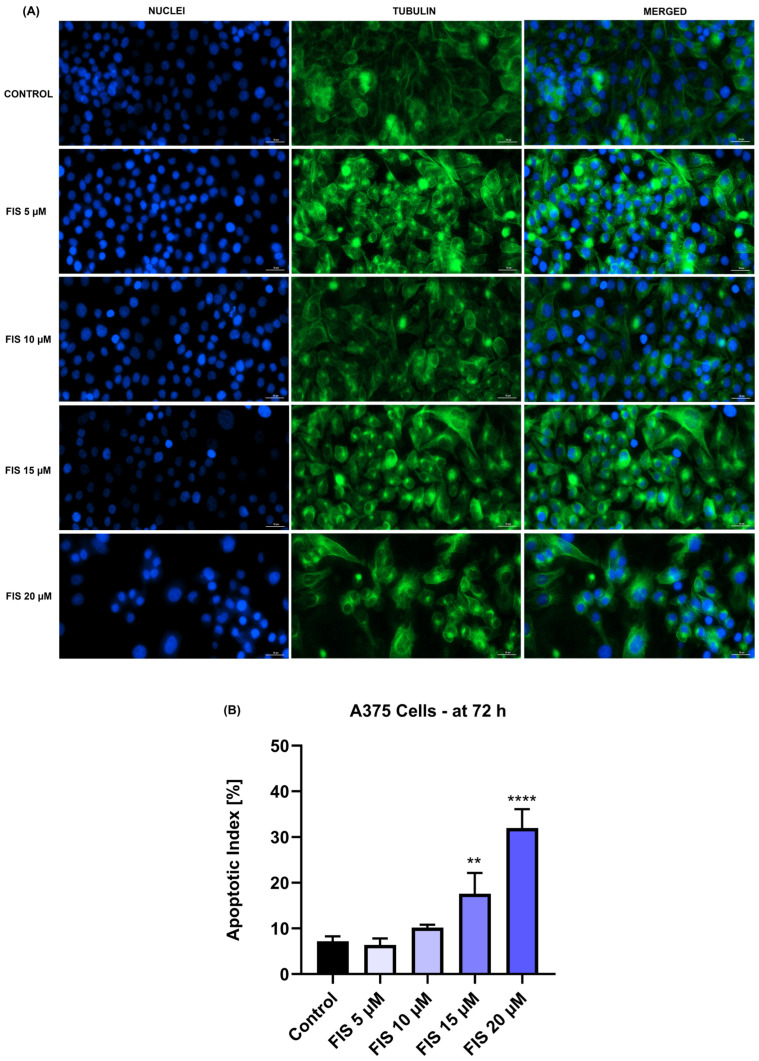
(**A**) Nuclei (blue) and tubulin filaments (green) of A375 cells treated for 72 h with fisetin (FIS) at four micromolar concentrations (5, 10, 15, and 20 µM). Scale bars show 30 µm. (**B**) Apoptotic index calculation in A375 cells treated for 72 h with fisetin (FIS) at four micromolar concentrations (5, 10, 15, and 20 µM). The results represent means ± standard deviation of three experiments done as triplicates. One-way ANOVA analysis and Dunnett’s multiple-comparison post-test were performed to determine the statistically significant differences between control and treatment (** *p* < 0.01; **** *p* < 0.0001).

As regards the FIS-only treatment, the most significant alterations in the nuclear aspect (e.g., massive condensation), tubulin organization (e.g., constriction and filament disruption), and apoptotic index were obtained at the highest concentration of 20 µM (Figure 6A,B).

**Figure 7:**
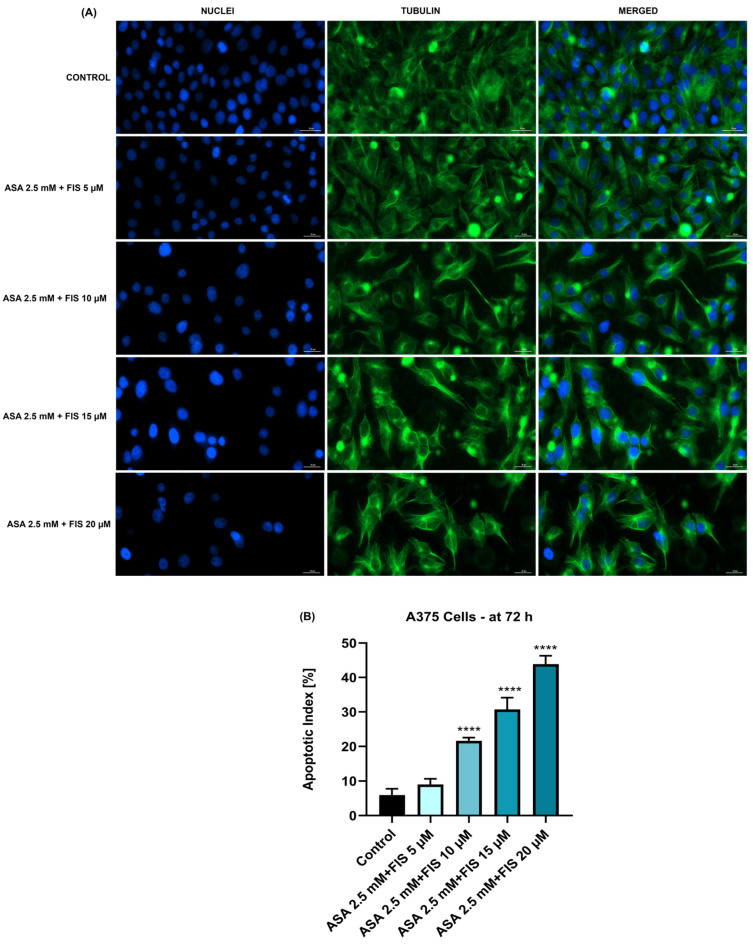
(**A**) Nuclei (blue) and tubulin filaments (green) of A375 cells treated for 72 h with the association between ASA 2.5 mM and FIS at four micromolar concentrations (5, 10, 15, and 20 µM). Scale bars show 30 µm. (**B**) Apoptotic index calculation in A375 cells treated for 72 h with the association between ASA 2.5 mM and FIS at four micromolar concentrations (5, 10, 15, and 20 µM). The results represent means ± standard deviation of three experiments done as triplicates. One-way ANOVA analysis and Dunnett’s multiple-comparison post-test were performed to determine the statistically significant differences between control and treatment (**** *p* < 0.0001).

The association between ASA 2.5 mM and FIS (5, 10, 15, and 20 µM) triggered the highest changes in nuclear shape and tubulin aspect as follows (Figure 7A): (i) ASA 2.5 mM + FIS 5 µM induced nuclear condensation and shrinkage while tubulin presented a distribution similar to control cells; (ii) ASA 2.5 mM + FIS 10 µM induced condensation of both cellular components; (iii) ASA 2.5 mM + FIS 15 µM caused a massive constriction of chromatin and tubulin associated with loss in cell contact and shape changing, A375 cells adopting a more spherical morphology; and (iv) ASA 2.5 mM + FIS 20 µM disrupted the tubulin fibers and produced chromatin condensation and nuclear dysmorphology. Figure 7B shows that these combinatorial treatments also elevated the apoptotic indexes in A375 cells, statistical significance being obtained for ASA 2.5 mM + FIS 10 µM, ASA 2.5 mM + FIS 15 µM, and ASA 2.5 mM + FIS 20 µM associations.

### 3.4. Influence of ASA, FIS, and ASA + FIS on Cell Migration

**Figure 8:**
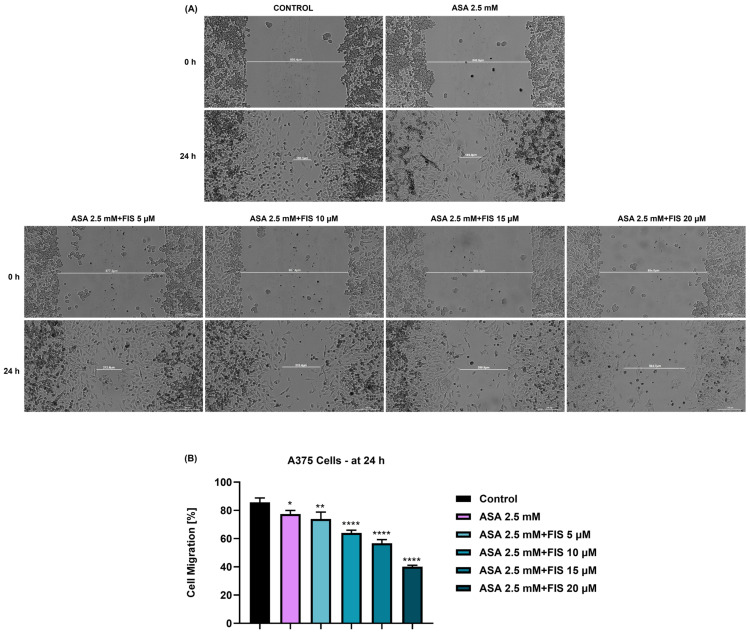
(**A**) Scratch closure in A375 cells treated for 24 h with ASA 2.5 mM, as well as with the association between ASA 2.5 mM and FIS at four micromolar concentrations (5, 10, 15, and 20 µM). Scale bars show 200 µm. (**B**) Graphical representation of migration percentages in A375 cells treated for 24 h with ASA 2.5 mM, as well as with the association between ASA 2.5 mM and FIS at four micromolar concentrations (5, 10, 15, and 20 µM). The results represent means ± standard deviation of three experiments done as triplicates. One-way ANOVA analysis and Dunnett’s multiple-comparison post-test were performed to determine the statistically significant differences between control and treatment (* *p* < 0.05; ** *p* < 0.01; **** *p* < 0.0001).

The potential anti-migratory activity of ASA, FIS, and ASA + FIS treatments was further investigated. FIS (5, 10, and 15 µM) was found to exert a stimulatory effect on the migration ability of A375 cells, while at 20 µM it had no influence on cell motility compared to control. Intriguingly, as illustrated in Figure 8, compared to control, in which the cell migration rate was approximately 80%, ASA 2.5 mM showed a slight inhibition to around 77%, while the combination of ASA 2.5 mM with FIS (5, 10, 15, and 20 µM) resulted in a concentration-dependent and statistically significant blockage of A375 cell migration. The cell migration percentages for ASA 2.5 mM + FIS 5 µM, ASA 2.5 mM + FIS 10 µM, ASA 2.5 mM + FIS 15 µM, and ASA 2.5 mM + FIS 20 µM were 73.9%, 64.0%, 56.70%, and 40.13%, respectively.

### 3.5. Irritant Potential of ASA, FIS, and ASA + FIS on the CAM

**Figure 9:**
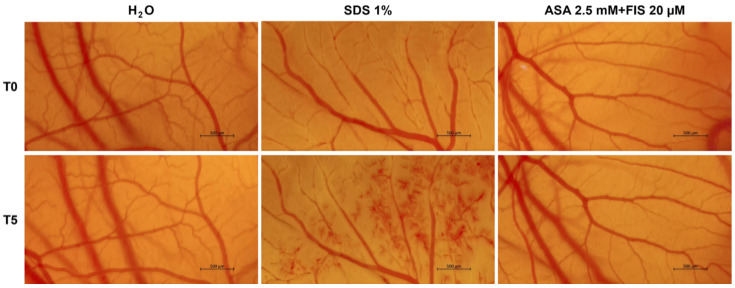
Representative images of the chorioallantoic membrane vasculature before (T0) and 5 min after (T5) the local application of H_2_O (negative control), sodium dodecyl sulphate (SDS) 1% (positive control), and the association between ASA 2.5 mM and FIS 20 µM. Scale bars show 500 µm.

The local irritant effect of the ASA 2.5 mM + FIS 20 µM treatment was assessed in ovo, using the CAM as biological model. As shown in Figure 9, the only changes in vascular architecture were caused by SDS 1% used as positive control, which induced lysis, coagulation, and hemorrhage immediately after its direct contact with the CAM blood vessels. H_2_O exerted no vascular toxicity, while the ASA 2.5 mM + FIS 20 µM combination induced only a slight coagulation in some microvessels at the end of the experiment.

Based on the obtained IS value, which was lower than 1 (Table 1), ASA 2.5 mM + FIS 20 µM was classified as non-irritant on the CAM. The only severe irritant potential was observed in the case of SDS 1%.

### 3.6. Influence of ASA, FIS, and ASA + FIS on In Ovo Angiogenesis

**Figure 10:**
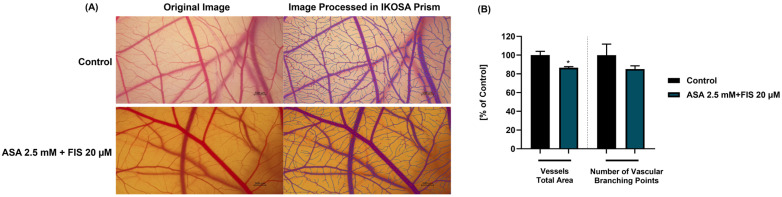
(**A**) Representative images (original and processed in IKOSA Prism) of CAM angiogenesis at 24 h after its exposure to the association between ASA 2.5 mM and FIS 20 µM. Control represents CAM without treatment. Scale bars show 500 µm. (**B**) Quantification of vessels’ total area and number of vessels’ branching points using the IKOSA Prism AI Cam Assay (V3.1.0). The unpaired *t* test was performed to determine the statistically significant differences between control and treatment. “*” indicates statistically significant reduction in vessels’ total area (* *p* < 0.05).

The potential angio-inhibitory effect of the ASA 2.5 mM + FIS 20 µM association was finally explored. As documented in Figure 10A,B, compared to control, this combinatorial treatment halted CAM angiogenesis, which was evidenced by a reduction in total vascular area and number of vascular branching points (to around 80%).

## 4. Discussion

MM is a severe cutaneous neoplasm, presenting a continuous rise in incidence worldwide [33]. At present, the existing treatment options for MM (i.e., surgery, chemotherapy, immunotherapy, targeted therapy, etc.) face numerous limitations that range from undesirable adverse reactions to tumor resistance and poor response to therapy, thus altering the outcomes for diagnosed patients [5,7]. Therefore, alternative treatment strategies are highly required and must be further researched. Recently, several novel approaches for MM management were identified as promising options for the management of this skin cancer subtype, as follows: (i) application of dietary compounds presenting an increased safety profile and multimodal anticancer activities in MM treatment [34]; (ii) drug repurposing and redirection of the currently marketed medicines towards MM treatment [19]; and (iii) utilization of combinatorial treatments for MM [35]. Considering these aspects, the present study proposed a comprehensive investigation of the combinatorial treatment between a dietary agent (FIS) and a candidate for drug repurposing (ASA) as a potential therapeutic solution for MM treatment. The investigations were performed using the A375 cell line as a representative model for MM due to its harboring of the BRAF^V600E^ mutation [36], and comparatively using the non-tumorigenic HaCaT immortalized cell line as a proper model for normal skin-derived cells owing to its high similarity to isolated keratinocytes [37]. The concentrations tested herein—ASA 1, 2.5, 5, and 7.5 mM; FIS 5, 10, 15, and 20 µM—were chosen to cover the ones that are physiologically achievable following the compounds’ administration [11,38]. Additionally, ASA 2.5 mM was further selected for combinatorial treatment with FIS (5, 10, 15, and 20 µM), considering not only its pharmacological relevance in clinical practice [39] but also its previously documented efficiency in enhancing the therapeutic efficacy of other anti-neoplastic agents [27,39,40].

The study was built upon previous reports highlighting the anti-melanoma effect exerted by individual treatments with either FIS or ASA. For instance, the anti-neoplastic potential of FIS in the treatment of cutaneous cancers was previously explored by Imtiyaz et al. The results of their study revealed that FIS induced dose-dependent cytotoxicity in two skin cancer cell lines with IC_50_ values of 57.60 µM ± 6.59 (at 24 h) and 41.70 µM ± 1.25 (at 72 h) in A375 cells, as well as 48.70 µM ± 5.49 (at 24 h) and 33.67 µM ± 1.03 (at 72 h) in A431 cells [41]. Pal et al. investigated the therapeutic efficacy of FIS in a concentration range of 5–20 µM for 24 h on three BRAF-mutated cell lines (i.e., A375, SK-MEL-28, RMPI-7951), the SK-MEL-119 NRAS-mutated cell line, and the NRAS-BRAF mutated HS294T cell line, their investigation concluding that BRAF-mutated MM cells show a higher sensitivity to FIS-based therapy. Additionally, the same study showed that FIS blocked the invasion of MM cells and suppressed the formation of nodes in 3D reconstructed human MM skin equivalents containing A375 cells [17]. Another study illustrated the selective cytotoxic effects of FIS, which caused a concentration-dependent reduction in the growth of metastatic 451Lu MM cells, while normal human melanocytes were resistant to its growth repression activity. The authors estimated that the IC_50_ values for FIS in this MM cell line were 80 µM (at 24 h), 37.2 µM (at 48 h), and 17.5 µM (at 72 h) [42]. As regards the anti-tumor properties of ASA in MM cell lines, several studies have deciphered its therapeutic potential. One such example would be the work of Fujikawa et al., who demonstrated that ASA (1.3, 2.5, 5, and 10 mM) exerts a preferential cytotoxicity in A375 and A2058 MM cells, compared to healthy HDF cells, after a treatment of 72 h [24]. Vad et al. showed that ASA exerted growth-suppressing effects in B16-F0 MM cells after 48 h of treatment, with an IC_50_ value of 100 μM [43]. The initial results obtained during this study regarding the cytotoxicity of ASA and FIS in MM cells completed these previous findings. The 72 h treatment of A375 cells with both ASA and FIS resulted in a concentration-dependent cytotoxic activity, the strongest effects being obtained at the highest tested concentrations (ASA 5 mM and 7.5 mM; FIS 15 µM and 20 µM), at which a reduction in cell viability (<50% for ASA and <95% for FIS) was observed, accompanied by the loss of cell confluence, changes in cell morphology, alterations in nuclear aspect and tubulin organization, and increased apoptotic index (Figure 1, Figure 3, Figure 5 and Figure 6).

As an element of novelty, this study presents one of the first assessments of the potential enhanced in vitro therapeutic activity against MM resulting from the association of ASA with FIS. The findings related herein demonstrated that the addition of ASA 2.5 mM to FIS (5, 10, 15 and 20 µM) leads to improved cytotoxic activity against A375 cells compared to the individual treatments, with determined viabilities of 89.33%, 82.86%, 61.24%, and 57.09% for ASA 2.5 mM + FIS 5 µM, ASA 2.5 mM + FIS 10 µM, ASA 2.5 mM + FIS 15 µM, and ASA 2.5 mM + FIS 20 µM associations (Figure 1), respectively. The selectivity of these combinatorial treatments towards MM cells and lack of toxicity in healthy skin-derived cells were also found herein, ASA + FIS exerting a low impact on the viability of HaCaT keratinocytes after 72 h of treatment (Figure 2). To the best of our knowledge, studies on the potential toxicity resulting from the association between ASA and FIS are currently scarce, although their individual side effects were mentioned in the literature. FIS is generally considered safe when consumed, although high supplementation might lead to gastrointestinal adverse reactions (e.g., nausea, vomiting, diarrhea). Additionally, FIS may also elevate the risk of bleeding associated with the administration of blood thinners [44]. Regarding ASA, its main toxic events include gastrointestinal impairments and hemorrhagic complications [45]. Therefore, considering that both FIS and ASA are associated with adversities at the gastrointestinal level and bleeding, the potential occurrence of these effects during their concurrent administration requires additional investigations.

Considering that apoptosis differs from other cell death mechanisms through specific morphological features (e.g., cell shrinkage, nuclear fragmentation, constriction, cytoskeletal rearrangements) [46,47], this study also assessed the impact of ASA + FIS combination therapy on A375 cells’ morphology, nuclear aspect, and tubulin organization. As presented in Figure 4 and Figure 5, all treatments comprising ASA 2.5 mM and FIS (5, 10, 15, and 20 µM) caused massive changes in cell shape, confluence, nuclear, and tubulin aspects; however, the greatest alterations (i.e., confluence loss, shrinkage, rounding, nuclear dysmorphology and condensation, tubulin constriction and disruption) were observed in the case of the ASA 2.5 mM + FIS 20 µM combination. One of the main issues leading to poor prognosis and survival rates among diagnosed patients is the high metastatic ability of MM. During invasion, MM cells lose cell-to-cell adherence, gain motility, and invade neighboring tissues, forming metastases. The presence of the BRAF^V600E^ mutation in MM cells was previously associated with an enhancement in the metastatic potential [17]. Therefore, another interest of this research was in finding out whether the ASA + FIS combinations can inhibit the migration of BRAF^V600E^-mutated A375 MM cells. As observed in Figure 8, the association of ASA 2.5 mM with FIS (5, 10, 15, and 20 µM) led to a dose-dependent and significant suppression of A375 cells’ migratory ability, the cell migration rates being 73.9%, 64.0%, 56.70%, and 40.13% for ASA 2.5 mM + FIS 5 µM, ASA 2.5 mM + FIS 10 µM, ASA 2.5 mM + FIS 15 µM, and ASA 2.5 mM + FIS 20 µM, respectively, compared to 80% for control. To the best of our knowledge, the enhanced therapeutic efficacy resulting from the association of ASA with FIS in the treatment of MM or other cancer types has received no recognition to date. However, preceding reports highlighted the efficacy of the combinatorial treatment between FIS/ASA and anti-tumor agents in cancer treatment. One such study illustrated the ability of FIS to potentiate the efficiency of the RAF inhibitor sorafenib to inhibit cell growth, block colony formation, and trigger apoptosis in three BRAF-mutated MM cells (i.e., A375, SK-MEL-28, and RPMI-7951) [48]. We have recently reported the efficacy of the combinatorial treatment between ASA 2.5 mM and GEN (10–75 µM) to cause massive cytotoxicity and exert anti-migratory properties in HCT-116 human colorectal carcinoma cells [11].

In addition to these in vitro findings, the study also aimed to extend the exploration of ASA + FIS treatment to in ovo investigations. Therefore, the potential irritant effect of this association between ASA and FIS, as well as its influence on CAM neovascularization, was finally explored. The HET–CAM test was applied herein for the evaluation of the local irritant potential of the ASA 2.5 mM + FIS 20 µM combination, considering its specific application in the assessment of the potential mucosal irritation and local toxicity of various formulations [49]. According to the results (Figure 9, Table 1), the CAM’s exposure to ASA 2.5 mM + FIS 20 µM triggered no vascular impairment apart from only slight signs of coagulation. Overall, this treatment was classified as non-irritant on the CAM, presenting an irritation score of 0.82. Angiogenesis has been well-documented as a crucial process in tumor growth and metastasis formation [50]. Previous reports have revealed both FIS and ASA as active agents in targeting angiogenesis and as angio-inhibiting compounds [51,52,53]. Additionally, this study assessed the anti-angiogenic potential of their association on the CAM, a pivotal biological model allowing the exploration of angiogenesis in response to various biomolecules or drugs due to its specific high-density vascular structure formed of blood and lymphatic vessels [50]. It was found that the treatment of the CAM with ASA 2.5 mM + FIS 20 µM resulted in an inhibition of neovascularization, observed through the reduction in the vessels’ total area and the number of vascular branching points (Figure 10). An angio-suppressing effect on the CAM was also obtained in the case of the combinatorial treatment comprising ASA 2.5 mM and genistein 50 μM [11].

## 5. Conclusions

The novel findings of the current study unveiled the combinatorial treatment between ASA and FIS as a potential complementary alternative option for the treatment of MM, as this association was found to exert significant cytotoxicity, induce apoptotic-like features, and suppress cellular motility in A375 BRAF^V600E^-mutated MM cells, while also presenting an anti-angiogenic activity on the CAM. This work stands as an initial preclinical evaluation of ASA + FIS for applications in MM therapy, opening a new research area for further investigations.

## Figures and Tables

**Table 1:** Irritation score (IS) determined following the local treatment of the CAM with H_2_O (negative control), sodium dodecyl sulfate (SDS) 1% (positive control), and the association between ASA 2.5 mM and FIS 20 µM.

Treatment	Calculated Irritation Score (IS)	Interpretation
H_2_O	0.07	Non-irritant
SDS 1%	19.51	Strong irritant
ASA 2.5 mM + FIS 20 µM	0.82	Non-irritant

## Data Availability

The data presented in this study are available on request from the corresponding author.
